# Bilateral spermatic cord en bloc ligation by laparoendoscopic single-site surgery: preliminary experience compared to conventional laparoscopy

**DOI:** 10.1186/1471-2490-14-83

**Published:** 2014-10-23

**Authors:** Salvatore Micali, Ahmed Ghaith, Eugenio Martorana, Alessio Zordani, Angelo Territo, Giampaolo Bianchi

**Affiliations:** Department of Urology, University of Modena & Reggio Emilia, Via del Pozzo, 71, Modena, 41124 Italy; Department of Urology, Tanta University, Tanta, Egypt

**Keywords:** Laparo Endoscopic Single-site Surgery (LESS), Transumbilical access, Bilateral spermatic cord ligation

## Abstract

**Background:**

Laparo Endoscopic Single-site Surgery (LESS) represents an evolution of minimally invasive surgery and aims to improve cosmetic outcome and reduce surgical trauma and complications associated with traditional laparoscopy. This study was performed to present our preliminary experience in bilateral spermatic cord ligation with the LESS technique and compare the results with the outcomes of conventional laparoscopic surgery.

**Methods:**

Between June 2007 and May 2013, 24 patients were referred to our institute for bilateral varicocelectomy. The indications for this type of procedure were bilateral varicocele with impairment of semen parameters or chronic bilateral testicular pain. All procedures were performed via the same surgeon. The patients were divided into two groups according to the type of laparoscopic surgery. Group A included 10 patients underwent LESS technique while group B included the remaining 14 patients that underwent conventional laparoscopy.

**Results:**

The comparison between the two techniques showed some important advantages for LESS: shorter operating time (45.4 min vs. 88.3 (P < .001), shorter hospital stay (16.6 hours vs. 51.4 hours) (P < .001), early return to the normal activity (2.3 days vs. 4.7 days) and better cosmetic outcomes. No conversions from LESS to conventional laparoscopy were necessary and blood loss was insignificant in all patients.

All patients in the LESS group reported full satisfaction with the cosmetic outcome, whereas 85.7% of patients after conventional laparoscopy were fully satisfied with cosmesis.

**Conclusions:**

Bilateral spermatic cord ligation with LESS is an alternative to conventional laparoscopy. The procedure was successfully performed in all patients. The trans-umbilical approach offers the advantage of a better cosmetic result, shorter hospital stay and less postoperative pain.

## Background

The prevalence of varicocele is approximately 15% to 20% in the general male population and it increases to 40% to 70% in men with primary and secondary infertility, thus making varicocele the most common correctable male infertility factor [1]. Less frequently, varicocele may be a source of chronic testicular pain.

There are several theories regarding the mechanism by which varicocele could inhibit the fertility potential of affected men. The most commonly proposed theory is that excess heat, due to the varicocele, reduces spermatogenesis. Some have also speculated that the effect is via reflux of metabolites into the testes [2, 3].

Various treatment modalities have been studied for patients with varicocele who have subfertility or scrotal pain, including percutaneous embolization, open (inguinal or high) varicocele ligation, subinguinal microsurgical varicocele ligation, and laparoscopic varicocele ligation. Each technique has advantages and disadvantages, and conflicting results have been achieved in different studies [4]. Advantages of laparoscopic varicocelectomy include: - increased magnification, facilitating more accurate identification of vessels [5, 6]. Also, laparoscopic varicocelectomy allows for en bloc and bilateral ligation of the spermatic cord.

Efforts to further reduce the morbidity and improve the cosmetic outcome of laparoscopic surgery have led to the evolution of Laparo-Endoscopic Single-site Surgery (LESS) [7]. LESS has been developed in an attempt to reduce the morbidity and multiple scarring associated with laparoscopic surgery [8]. The feasibility of LESS varicocelectomy as a treatment modality has been described [9].

In this study we present our preliminary experience of bilateral en bloc spermatic cord ligation with LESS technique and compare the results with the outcomes of conventional laparoscopic surgery.

## Methods

### Patient demographics

Between June 2007 and May 2013, 24 patients were referred to Urology Department of Modena University for bilateral varicocelectomy. The indications for this type of procedure were bilateral varicocele with impairment of semen parameters or chronic bilateral testicular pain, based on physical examination, semen analysis and Doppler ultrasonography. Bilateral varicocelectomy in our institute is a part of routine/standard care whether open or laparoscopic. All patients gave written informed consent for the technique. The patients were divided into two groups according to the type of laparoscopic surgery and the data was collected retrospectively.

Group A included 10 patients that underwent bilateral LESS en bloc spermatic cord ligation. Their age ranged from 24 to 44 years old (mean age: 30.2 years). Two cases were bilateral grade 2, one case was grade 4 on the left and grade 2 on the right side, one case was grade 3 on the left and grade 2 on the right side, and six cases were bilateral grade 3. Two of these patients had recurrence after previous left open varicocelectomy. One patient had chronic testicular pain [Table 1]. Group A patients were informed that such elective conversion to standard laparoscopy was not a complication but rather surgical prudence because of the novelty of this procedure.

**Table 1:** **LESS group patients’ characteristics**

Patient	Name	Age	Varicocele grade	Previous varicocelectomy
1	MG	24	Grade 4 on the left, grade 2 on the right side	No
2	GD	29	Bilateral grade 2	No
3	MM	29	Bilateral grade 3	No
4	AM	30	Bilateral grade 2	No
5	PC	31	Bilateral grade 3	No
6	LU	25	Bilateral grade 3	Left varicocelectomy
7	MS	44	Bilateral grade 3 with chronic testicular pain	Left varicocelectomy
8	AG	32	Bilateral grade 3	No
9	EM	30	Bilateral grade 3	No
10	SM	28	Grade 3 on the left and grade 2 on the right	No

Group B included the remaining 14 patients that underwent bilateral varicocelectomy using conventional laparoscopy. The age ranged from 17 to 42 years old (mean age: 29.3 years), two cases were bilateral grade 3, four cases were bilateral grade 2, one case was bilateral grade 1, four cases were left grade 2 and right grade 1, two cases were left grade 3 and right grade 2 and one was left grade 4 and right 2. Three of these patients had recurrence after previous left open varicocelectomy. Two patients had chronic testicular pain [Table 2].

**Table 2:** **Conventional laparoscopy group patients’ characteristics**

No.	Name	Age	Varicocele grade	Previous varicocelectomy
1	PL	20	Bilateral grade 2	No
2	PS	41	Left grade 4	Left varicocelectomy
			Right grade 2	
3	GM	22	Left grade 3	No
			Right grade 2	
4	YO	30	Left grade 2	Left varicocelectomy
			Right grade 1	
5	YS	18	Bilateral grade 2	No
6	AA	19	Left grade 3	No
			Right grade 2	
7	DS	17	Bilateral grade 2	No
8	TT	42	Bilateral grade 2	No
9	PS	30	Left grade 2	No
			Right grade 1	
10	RD	41	Left grade 2	No
			Right grade 1	
11	MA	20	Bilateral grade 3	No
12	RP	38	Left grade 2	Left varicocelectomy
			Right grade 1	
13	AM	39	Bilateral grade 1	No
14	BC	33	Bilateral grade 3	No

Transabdomenal sonography was done in all cases to exclude retroperitoneal masses and preoperative semen analysis was performed in all patients. Exclusion criteria included patients who underwent previous abdominal surgery. All patients provided informed consent.

All procedures were performed in the Urology Department of the University of Modena and Reggio Emilia, Italy. Laparoscopic and LESS varicocelectomies were performed by an individual urologist who was skilled in and experienced with laparoscopic surgery using a standardized protocol.

All data were collected retrospectively in excel file without any patient identifying information. Since each patient signed a general consent to the processing of personal data it was not necessary the institutional review board’s approval for the data.

### Operative technique

**Figure 1:**
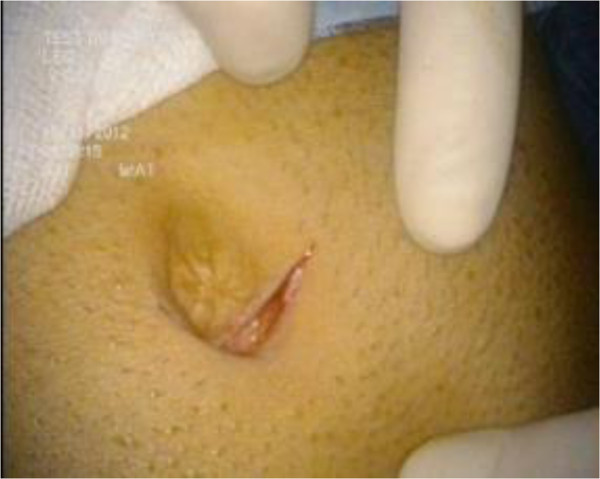
**Umbilical incision.**

**Figure 2:**
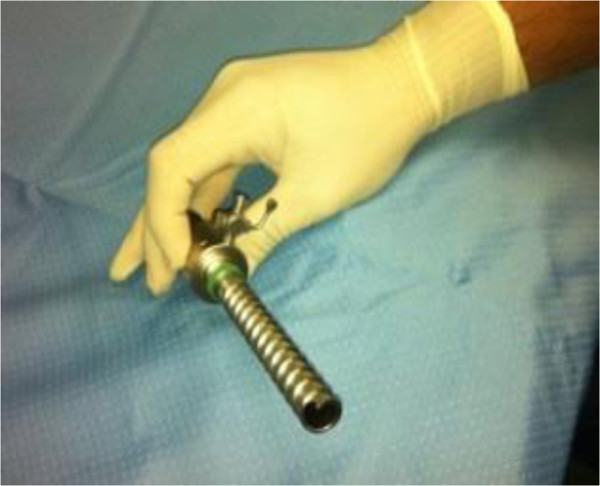
**Ternamian EndoTIP 10 mm optical trocar.**

**Figure 3:**
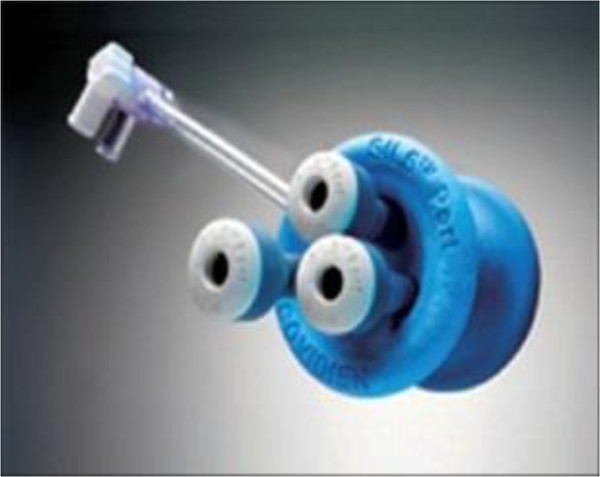
**Covidien SILS™ port.**

After insufflation with carbon dioxide at 15 mmHg, the procedure started with 2 cm incision on the lower margin of the umbilicus (Figure 1). In the first five cases our transperitoneal access was created with the aid of 10 mm optical laparoscopic visual reusable trocar, Ternamian EndoTIP 10 mm (Karl Storz®, Tuttlingen, Germany) (Figure 2). This trocar enabled dissection of each tissue layer under direct vision, so that the surgeon has the visual control needed to avoid blood vessels. The next step was to substitute the optical trocar with a disposable multiport trocar (Covidien SILS™ Port, Mansfield, MA, USA) (Figure 3).

**Figure 4:**
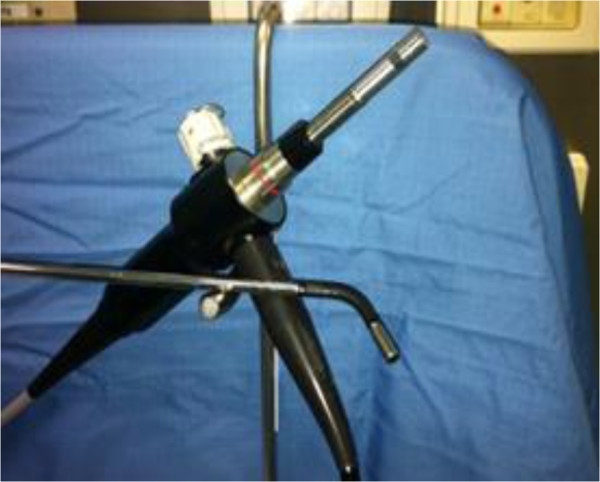
**EndoEye camera system.**

In the next five cases we performed an open technique to access the abdominal cavity and position the SILS port. Trans-umbilical access has two advantages: the use a pre-existing scar gives better cosmetic result, and trans-umbilical access allows for quick identification of the spermatic cords and vas deferens bilaterally by the same incision. The SILS port allows the insertion of a 5-mm flexible laparoscope EndoEye camera system (Olympus Medical, Orangeburg, NY, USA) that minimizes the internal and external clashing of the instruments (Figure 4). Standard, reusable, 5 and 10 mm laparoscopic instruments were used to perform the procedure.

A 3–4 cm “T” incision of the posterior parietal peritoneum was done lateral to the spermatic cord and proximal to the internal ring and vas deferens. The spermatic cord was isolated en bloc and four Hem-o-lok® (Teleflex Medical Research, Triangle Park, NC, USA) were placed for haemostasis. It is important to ensure vas deferens preservation with its artery to ensure blood supply to the ipsilateral testis. The same manoeuvre was then performed on the other side. At the end of the procedure the abdominal cavity is slowly deflated for haemostasis control.

### Conventional laparoscopic varicocelectomy

The surgical steps were identical to those described for LESS technique. Under general anesthesia the patient is positioned supine and a Foley catheter is placed in the bladder. The abdomen is insufflated to 15 mm. Hg and a 12 mm transumbilical optical trocar was used for the 0-degree lens. A second 5 mm. port and an additional 10 mm. port were placed in the mid line of lower abdomen between umbilicus and symphysis pubis and used for dissection using standard, reusable, 5 and 10 mm laparoscopic instruments.

The overlying peritoneum is incised and the spermatic cord is isolated circumferentially proximal to the internal inguinal ring and the vas deferens.

Selective bilateral varicocelectomy was performed in 5 cases. Gonadal vein is identified and adjacent tissue with lymphatics is swept away, and the vein is ligated using titanium clips. The papaverine test is used for identification and preservation of the testicular artery. En bloc ligation of the spermatic cord and surrounding tissue was performed in the remaining 9 cases using Hem-o-lok®.

## Results

All procedures were performed successfully. Table 3 summarizes the results of both groups.

**Table 3:** **Results**

	LESS varicoselectomy	Convention laparoscopic varicocelectomy
Number of patients	10	14
Mean operative time	45.4 minutes	88.3 minutes
Mean blood loss	< 30 ml	< 30 ml
Mean hospital stay	16.6 hours	51.4 hours
Postoperative analgesia	0	5 patients
Mean time return to work	2.3 days	4.7 days
Technique	Non selective	5 patients: selective
		9 patients: non-selective
Complications	No	2 patients

In LESS group operative time ranged from 31 to 75 minutes (mean: 45.4 minutes), and the estimated blood loss was insignificant. The hospital stay ranged from 12 to 24 hours (mean: 16.6 hours). Time to return to work ranged from 2 to 3 days (mean: 2.3 days). None of the patients required narcotics or additional analgesia in the postoperative period. No intraoperative nor postoperative complications occurred.

In conventional laparoscopy group operative time ranged from 65 to 130 minutes (mean: 88.3 minutes), and the estimated blood loss was insignificant. The hospital stay ranged from 24 to 168 hours (mean: 51.4 hours). Time to return to work ranged from 4 to 7 days (mean: 4.7 days). Five patients required postoperative analgesia. Two patients had lefts orchitis and one of these developed an abdominal hematoma.

A scrotal echo-color Doppler was performed after 3, 6 and 12 months in all patients of both groups and showed no recurrence of varicocele, no testicular hypotrophy nor secondary hydrocele developed in any patients. A postoperative semen analysis was done 3, 6 and 12 months post-procedure to evaluate the sperm concentration, motility, and morphology; it showed an improvement of semen parameters in seven of LESS group patients versus nine patients of the other group. The patients with chronic testicular pain in both groups had improvement after 3 months. Six months after the procedure, the patients were completely pain free.

**Figure 5:**
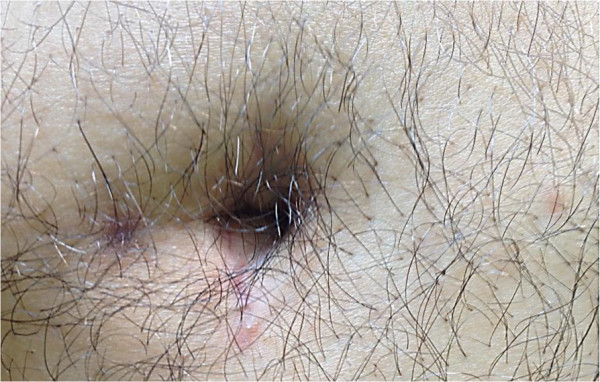
**Postoperative umbilical scar.**

All patients in the LESS group reported full satisfaction with the cosmetic outcome (Figure 5), whereas 85.7% of patients after conventional laparoscopy were fully satisfied with cosmesis.

The comparison between the two techniques showed some important advantages for LESS: shorter operating time (45.4 min vs. 88.3 (P < .001), shorter hospital stay (16.6 hours vs. 51.4 hours) (P < .001), early return to the normal activity (2.3 days vs. 4.7 days) and better cosmetic outcomes. No conversions from LESS to conventional laparoscopy were necessary and blood loss was insignificant in all patients.

## Discussion

Varicocele is considered one of the most important causes of male infertility and prepubertal testicular hypotrophy. The most common indications for varicocelectomy are subfertility and chronic orchialgia [10, 11]. Many clinical trials report a beneficial effect of varicocele repair on male reproduction with improvement in semen parameters, but some show the contrary [12].

The ideal method of varicocele treatment is a controversial issue. Several methods have been used, including open surgical ligation of the spermatic vein, retrograde or antegrade sclerotherapy, and laparoscopic and microsurgical varicocelectomy. Each technique has advantages and disadvantages, and conflicting results have been achieved in different studies [13].

The role of laparoscopic varicocelectomy is still controversial. According to our experience, the laparoscopic approach should be considered mostly in patients requiring bilateral varicocelectomy, since it appears to be a sensible alternative to 2 groin incisions. Also laparoscopic approach has short operative time and offers an easier postoperative course.

LESS has been developed in an attempt to further reduce the multiple ports morbidities and scarring associated with laparoscopic surgery [8]. In many studies, LESS varicocelectomy was reported to have acceptable feasibility, high patient satisfaction, and good postoperative outcomes [8, 9, 14–16]. Our study is in correspondence with these previous studies. It demonstrates that bilateral LESS varicocele ligation provides the best results comparison with those of the conventional laparoscopic technique.

The mean operative time was shorter in LESS group than in conventional laparoscopic group (45.4 minutes vs 88.3 minutes). The longer operative time in conventional laparoscopy group may be related to multiple numbers of access and performance of selective varicocelectomy in 5 of these patients. Dong Hyuk Kang et al., compared postoperative outcomes in patients treated with selective and non-selective LESS varicocele ligation. Mean operative time in non-selective group was similar to ours (48.6 minutes) while the mean operative time of selective group is shorter than that we found in conventional laparoscopy (60.7 minutes) [17]. This finding clarifies that the longer operative time in conventional laparoscopy may be related principally to multiple numbers of access.

The benefits of minimally invasive surgery are well recognized and include shorter length of stay, less analgesic requirement, faster convalescence, and better cosmetic outcomes. LESS is emerged to encounter these advantages. Our study showed that hospital stay and return to work were shorter in LESS group (16.6 hours vs 51.4 hours and 2.3 days vs 4.7 days respectively).

We used an optical trocar to do transperitoneal access in the first five cases but, we found that it makes a false subcutaneous plain, as it has a smooth end and the subcutaneous fascia at the umbilicus is tough. This optical trocar technique prolonged operative time, and as a result we decided to directly perform open access to enter the abdominal cavity in the remaining five cases.

In our experience, using Covidien SILS™ Port gives many advantages. It is an excellent device that can be handled easily. It is composed of two parts, a sponge cork and three trocars. The sponge cork will seal the skin incision and the trocars prevent CO2 leakage which is considered as an advantage over a gel port, which usually leaks. The optic with which we have obtained the best results is the 5-mm flexible laparoscope EndoEye camera system (Olympus Medical). This device provides flexibility and better working conditions [8]. In our experience we placed the instruments on different levels in the space with camera; with this expedient, the contact or clashing of instruments and the camera during LESS can be avoided.

Using transumbilical access, in LESS varicocelectomy, which is a pre-existing scar has potential better cosmetic outcomes and patient’s satisfaction. Also, it allows for bilateral en bloc ligation of the spermatic cord through the same access.

All patients underwent LESS vaicocelectomy showed better cosmetic outcomes and were very satisfied while 85.7% of patients underwent conventional laparoscopy were very satisfied by cosmetic outcomes.

All patients who underwent LESS varicocelectomy in our study did not need postoperative analgesia while postoperative NSAIDs were used in 35.7% of patients underwent conventional laparoscopy. This corresponds with the results of Xue et al. who performed a prospective, randomized study comparing LESS with conventional laparoscopic varicocele ligation. They reported that the visual analog scale (VAS) pain score was significantly lower 6 and 24 hours after surgery in patients who underwent LESS [8].

Our principle is en bloc ligation of the spermatic cord proximal to the internal inguinal ring via LESS technique for treatment of patients with varicocele to preserve the distal gonadal artery flow via collaterals from the proximal deferential artery. The effect of artery preserving varicocele ligation is still controversial, as the testicles receive arterial supply mainly from the testicular artery, supplemented by the cremasteric and vasal arteries [18].

The division of the spermatic vessels for difficult orchiopexy was suggested by Bevan in 1903. Later, Fowler and Stephens described the anatomy that allowed division of the spermatic vessels to gain additional length and bring the testis to the scrotum while maintaining collateral blood supply [19, 20]. Yamamoto et al. compared the two surgical methods and found no significant difference between testicular artery preservation and ligation varicocelectomy regarding semen quality, pregnancy rates, or testicular volume [21].

The retroperitoneal en bloc high ligation of a varicocele has the advantage of a lower incidence of recurrence due to ligation of the entire spermatic cord, including the artery and periarterial plexus of fine veins (venae comitantes), which may present as the source of recurrence [22].

The results of these studies correspond with our results. All patients included in both groups performed scrotal Doppler US at 3, 6, and 12 months and showed no recurrence of varicocele, no postoperative testicular atrophy nor secondary hydrocele.

In contrast, Zampieri et al. found that those with artery- sparing had better postoperative semen parameters than those who had undergone en bloc ligation that included the artery [23]. Raman and Goldstein also recommended preserving the testicular arteries for optimal testicular blood flow. However, prior series have revealed increased recurrence using the artery sparing method vs. taking the artery and veins [24, 25].

The limitations of our study are the relatively small number of patients. Large randomized prospective trials are required.

## Conclusions

LESS is an effective technique for various urology indications. LESS en bloc bilateral spermatic cord ligation is safe, effective, and showed good results in terms of intraoperative outcomes, postoperative improvement in pain and semen parameters, and patient satisfaction, with no major complications. Minimal hospitalization requirement following LESS spermatic cord ligation is an additional timely advantage over conventional laparoscopic and open surgeries.

## Abbreviations

VAS: Visual analog scale
LESS: Laparo endoscopic single site surgery.
